# Personal Identity After an Autism Diagnosis: Relationships With Self-Esteem, Mental Wellbeing, and Diagnostic Timing

**DOI:** 10.3389/fpsyg.2021.699335

**Published:** 2021-07-30

**Authors:** Kirsten Corden, Rebecca Brewer, Eilidh Cage

**Affiliations:** ^1^Department of Psychology, School of Life Sciences and the Environment, Royal Holloway, University of London, Egham, United Kingdom; ^2^Department of Psychology, Faculty of Natural Sciences, University of Stirling, Stirling, United Kingdom

**Keywords:** autism spectrum conditions, autism diagnosis, autistic identity, self-esteem, psychological wellbeing

## Abstract

Adults are increasingly seeking autism diagnoses, although less is known about their experiences of diagnosis and personal identity (i.e., autism as part of “me”), and how this relates to self-esteem and wellbeing. One-hundred and fifty-one autistic adults completed an online survey including measures of self-esteem, psychological wellbeing, and autistic personal identity, which considered whether participants took pride in or were dissatisfied with being autistic. Fifty-four participants answered a qualitative question about the impact of receiving an autism diagnosis on their sense of self. Regression analyses found that greater time elapsed since diagnosis related to less dissatisfaction with autistic personal identity. We also found that more dissatisfaction with autistic personal identity predicted lower self-esteem, and more autism pride predicted higher self-esteem. Content analysis of participants’ experiences supported the quantitative findings and was suggestive of an emotive post-diagnostic adjustment process. Future research should aim to identify ways to promote the development of a positive autistic personal identity post-diagnosis in adulthood.

## Introduction

Autism is a life-long neurodevelopmental condition characterised by differences in social communication and interactions, alongside sensory sensitivities, focused interests, and repetitive behaviours (DSM-5; American Psychiatric Association, 2013). Autism is often perceived as a childhood condition, with diagnostic criteria, protocols and service provision tailored to this age group (Howlin, 2008; Gerhardt and Lainer, 2011). However, many people receive an autism diagnosis in adulthood – something which is noted as emotionally impactful, with a notable lack of appropriate post-diagnostic support for adults (Huang et al., 2020). In the disability literature, acquiring a condition later in life necessitates accommodating changes into one’s self-concept (Frank, 1993). This process differs according to whether a well-formed identity has already been developed and committed to, known as achieving identity synthesis (Charmaz, 1994, 1995). Although autism is not an acquired condition, the impact of a diagnosis in adulthood, especially if the individual did not previously self-identify as autistic, could pose similar challenges to identity processes. Therefore, the current study firstly aimed to understand how autistic identity and diagnostic timing related to one another.

Social Identity Theory proposes that an individual’s self-concept is comprised of social and personal identities (Turner et al., 1987). Here, personal identity includes characteristics that define the individual and differentiate them from others. Personal autistic identity would encompass an individual’s own specific interests and values as an autistic person, which they perceive to contribute to their uniqueness and individuality (i.e., characteristics of “I” and “me”). Social identity, contrastingly, represents characteristics shared with a group someone identifies with. Autistic social identity would involve perceived similarities or shared characteristics with other autistic people (i.e., characteristics of “we” and “us”) and differentiation from out-groups (e.g., neurotypical people). The present study focused specifically on autistic *personal* identity.

Qualitative research with autistic adults indicates that identity formation may be challenging for those diagnosed later in life. In a study of autistic students’ experiences, individuals who received their diagnosis earlier in life expressed more acceptance of being autistic and a more positive sense of self (Cox et al., 2017). Other qualitative work with late-diagnosed older autistic adults (aged over 50) identified how some participants tended to externalise and view autism as separate to themselves, suggesting that they were not incorporating autism into their personal identity (Hickey et al., 2018). One interview study with late-diagnosed autistic females described an emotionally difficult adjustment period, but how diagnosis helped them to make sense of their identity (Leedham et al., 2020). Receiving an earlier diagnosis may link with more positive self-concepts, but it is unclear whether this relates to the age at diagnosis, or the time elapsed since diagnosis for understanding and adaptation. As such, the present study aimed to examine the relationships between personal identity, age of diagnosis, and recency of diagnosis as distinct variables.

Ideally, the process of positive personal identity development should culminate in an understanding, acceptance, and appreciation of the whole self (Gill, 1997). However, it is thought that having a disability or condition (including autism) can relate to challenges in developing a positive self-concept (Gill, 1997), and thus there are complex relationships between identity and psychological health. These complexities may partly link to being or feeling “different,” or being treated or stigmatised as such by others (Milton and Sims, 2016; Richards, 2016). Factors contributing to minority stress (e.g., discrimination and internalised stigma) might be internalised into a negative sense of personal identity (Tantam, 1992; Wright et al., 2000; Botha and Frost, 2020). This notion links to Theory of Social Stigma of Goffman (2009) which suggests that certain labels hold the power to “spoil” an individual’s identity, leading to ostracization from society and disruption of identity development processes.

As indicated, making sense of one’s identity is psychologically demanding, and therefore identity processes may relate to psychological variables such as self-esteem and mental wellbeing. Mental wellbeing refers to a broad concept covering both positive and negative aspects of mental health, such as feelings of depression and being able to cope with these (Maitland et al., 2021). Self-esteem is an evaluative attitude toward the self, indicating self-worth (Luhtanen and Crocker, 1992), and is associated with concepts such as optimism and self-confidence (Rosenberg et al., 1995; Lyubomirsky et al., 2005). Studies have noted both lower mental wellbeing and self-esteem in autistic adults compared to the non-autistic population (Nguyen et al., 2020; Maitland et al., 2021). However, positive relationships have been observed between self-esteem and social identity in non-autistic samples (Greenaway et al., 2015; Jetten et al., 2015). Accordingly, Cooper et al. (2017) found that having a stronger sense of autistic social identity was associated with more positive self-esteem, and noted that greater autistic social identification could link to better mental health *via* increased self-esteem. However, less is known about the role of autistic personal identity (i.e., autism as part of “me”) in autistic adults and how it relates to self-esteem and mental wellbeing. Given the high rates of emotional distress and diagnosable mental health conditions which have been reported in autistic individuals (Stewart et al., 2006; Gillott and Standen, 2007; Eaves and Ho, 2008; Lever and Geurts, 2016; Lai et al., 2019), it is important to look at potential contributors, such as identity, to autistic people’s mental wellbeing and self-esteem.

Overall, the current study aimed to examine aspects of personal identity for autistic people, with a specific focus on those diagnosed in adulthood. First, the study aimed to investigate relationships between personal autistic identity and diagnostic timing, specifically the age and recency of diagnosis, to understand how receiving a late diagnosis relates to personal identity development processes. Second, this study aimed to investigate the relationship between autistic personal identity and psychological health (self-esteem and wellbeing), as has been documented with autistic social identity (Cooper et al., 2017; Maitland et al., 2021), to examine how personal identity processes may link to self-esteem and wellbeing in late-diagnosed autistic adults.

We hypothesised that: (1) younger age of diagnosis and greater time elapsed since diagnosis would relate to more positive autistic personal identity and (2) more positive autistic personal identity would relate to higher levels of self-esteem and wellbeing. We also used qualitative methods to gather further information on individuals’ perceptions of how an autism diagnosis affected their sense of self. This mixed methods approach was deemed appropriate, with a focus on developing a deeper understanding of autistic people’s experiences of identity after receiving a diagnosis, while also enabling a larger sample than purely qualitative studies. Although there have been qualitative studies which highlight the potential impact on identity of receiving an autism diagnosis, particularly in adulthood (e.g., Bargiela et al., 2016; Hickey et al., 2018; Stagg and Belcher, 2019; Leedham et al., 2020), this study adds to this literature with our mixed methods approach and is novel in looking quantitatively at relationships with psychological health.

## Materials and Methods

### Participants

One-hundred and fifty-one participants from the United Kingdom took part. We included only participants from the United Kingdom since diagnostic processes and barriers may differ by country (Huang et al., 2020). One hundred and seventeen participants identified as cisgender female (77.6%), with 30 cisgender male (19.7%) and four non-binary or transgender participants (2.7%). Participants’ age ranged from 18 to 65 years old, with a mean of 31.26 (*SD* = 10.23). Further participant characteristics are available in Table 1, indicating that most participants were White British, educated to degree level, and in employment.

**Table 1:** Demographic characteristics of the study participants.

Variable	Categorical response	%
Ethnicity	White British	72.8
	Mixed or multiple ethnic groups	15.2
	Black British	7.3
	Asian British	4.0
	Other	0.7
Education	GCSEs or equivalent	8.6
	Apprenticeship	6.0
	A-Levels or equivalent	24.5
	Undergraduate degree	36.4
	Postgraduate degree	14.6
	Doctoral degree	8.6
	Prefer not to say	1.3
Employment^*^	Employed full-time	29.1
	Employed part-time	24.5
	Student	12.6
	Unable to work	9.9
	Unemployed	9.9
	Self-employed	6.6
	Carer	5.3
	Retired	2.0

* Denotes that participants could select multiple options.

Participants were required to have a formal autism diagnosis since the study considered the experience of receiving a diagnosis. Eighty-six participants (57.0%) self-reported a diagnosis of “Autism Spectrum Condition,” with the remaining 65 participants (43.0%) reporting “Asperger’s Syndrome.” As this information was self-reported, the Ritvo Autism and Asperger Diagnostic Scale (RAADS-14; Eriksson et al., 2013) was administered to validate the presence of diagnosable autistic characteristics. All participants scored above the RAADS-14 cut-off score of 14. Participants predominantly received their diagnosis through NHS services (96.0%), with few receiving their diagnosis privately (4.0%). Participants received their diagnoses between 2000 and 2020, reporting ages at diagnosis ranging from 6 to 62 years old (mean = 26.42, *SD* = 11.18, 78.8% diagnosed over the age of 18). On average, participants had received their diagnosis 4.95 years previously (*SD* = 4.09, range 0–20). Over half indicated they had additional neurodevelopmental or mental health diagnoses (58.3%).

Participants were recruited online using snowballing methods, with adverts posted on public and private social media (e.g., Facebook and Twitter) and sent directly to relevant autism groups, organisations, and charities between December 2019 and March 2020. Although we were particularly interested in the experiences of those diagnosed late, the survey was open to all. This study was reviewed and approved ethically by the Research Ethics Committee at Royal Holloway, University of London. All participants provided informed consent prior to participation.

### Materials and Procedure

We used “Qualtrics” as the online survey platform. Participants first completed questions concerning demographics and diagnoses. Due to variation in language preferences within the autistic community (Kenny et al., 2016), participants were given the opportunity to customise the survey to reflect their preferred terminology (“autistic person,” “person with autism,” or no preference). Accordingly, we presented participants with subsequent questions using either identity-first (64% preferred) or person-first (2%) language, or a combination if no preference (34%). Participants then completed the four measures in the order outlined and an optional qualitative question (see below).

#### Ritvo Autism and Asperger Diagnostic Scale

The RAADS is a 14-item screening tool for identifying autistic characteristics. Participants responded using a four-point Likert scale [“never true” (0) to “true now and when I was young” (3)], indicating duration of each symptom or experience, with a total possible score between 0 and 42 (Eriksson et al., 2013). Example items include “It is very difficult for me to work and function in groups.” Internal reliability of the RAADS-14 in the current study was acceptable (*α* = 0.60).

#### Rosenberg Self-Esteem Scale

We used the Rosenberg Self-Esteem Scale (RSE) to measure self-esteem, which includes 10 items scored on a four-point Likert scale [“Strongly Disagree” (1) to “Strongly Agree” (4)], with a total possible score from 10 to 40, with higher scores indicating higher self-esteem (Rosenberg, 1965). Example items include “I feel I have a number of good qualities.” Previous studies have demonstrated excellent internal reliability when used with autistic adults (e.g., Cooper et al., 2017; *α* = 0.91), and internal reliability in the current study was also excellent (*α* = 0.90).

#### Warwick-Edinburgh Mental Wellbeing Scale

The Warwick-Edinburgh Mental Wellbeing Scale (WEMWBS) is a 14-item measure of mental wellbeing. Items were rated on a five-point Likert scale [“none of the time” (1) to “all of the time” (5)], with a total possible range of 14–70, with higher scores indicating more positive mental wellbeing (Tennant et al., 2007). Example items include “I’ve been feeling useful.” Past research has shown the WEMWBS has excellent internal reliability with autistic adults (e.g., Cai et al., 2019; *α* = 0.90). Internal reliability in the current study was very good (*α* = 0.89).

#### Questionnaire on Disability Identity and Opportunity

We measured autistic personal identity using two subscales adapted from the Questionnaire on Disability Identity and Opportunity (QDIO; Darling and Heckert, 2010). This measure was selected as it reflected orientations and self-identification with a disability or condition. In our study, the word “disability” was substituted by the word “autism/autistic” as appropriate. The two subscales of interest were autism pride, reflecting perceived importance of or pride in autism being part of oneself, and exclusion/dissatisfaction, capturing feelings of rejecting or being dissatisfied with being autistic. Each subscale consisted of four items [e.g., “Autism is an important part of who I am” (autism pride); “Autism limits my social life” (exclusion/dissatisfaction)]. Participants scored each item on a five-point Likert scale [“Strongly disagree” (1) to “Strongly agree” (5)], with total possible scores ranging between 4 and 20 for each subscale. For autism pride, a higher score would indicate more pride in being autistic, and for exclusion/dissatisfaction, a higher score indicated more dissatisfaction with being autistic. In the original study, the authors reported good overall reliability levels (exclusion/dissatisfaction *α* = 0.73, disability pride *α* = 0.78). In this study, internal reliability was very good for autism pride (*α* = 0.84) and acceptable for exclusion/dissatisfaction (*α* = 0.68).

#### Qualitative Question

Participants could optionally provide qualitative information on the impact of receiving an autism diagnosis on their sense of identity. This question was presented prior to completion of the QDIO, to ensure qualitative responses were not influenced by the QDIO items: “*How did receiving an autism diagnosis affect how you think and feel about yourself? If possible, please refer to the following two periods of time: (a) When you initially received your diagnosis (b) The present day (i.e., currently)*.” The exact wording of the question was discussed with members of the autistic community to ensure clarity and acceptability.

### Design

This study used a cross-sectional mixed methods survey design. We selected mixed methods approaches to maintain a high level of empiricism while also acknowledging the exploratory nature of the study. Including qualitative elements in autism research has been highlighted as particularly important in understanding issues from the perspective of autistic people themselves (e.g., Bölte, 2014).

### Data Analysis

We analysed quantitative data using SPSS version 27. There were no missing data for any of the quantitative measures. We first calculated descriptive statistics and variables were tested for compliance with standard parametric assumptions. All *z* scores calculated for skewness and kurtosis were lower than 2.58 (*p* > 0.01), and therefore we considered the data to be normally distributed. We consider *p* values between 0.05 and 0.005 as suggestively significant and *p* < 0.005 as significant (Ioannidis, 2018).

For the first hypothesis (personal autistic identity, age of diagnosis, and recency of diagnosis), we conducted two separate multiple regressions with personal autistic identity (QDIO subscales) as the dependent variables, and age of diagnosis and recency of diagnosis as predictor variables (controlling for gender). These analyses aimed to show how diagnostic timing related to personal autistic identity, over and above any gender differences in diagnosis.

For the second hypothesis (autistic personal identity, self-esteem, and wellbeing), we used two hierarchical regression analyses. The first hierarchical regression had self-esteem (RSE scores) as the outcome variable. We entered control variables into the first step, specifically autistic characteristics (RAADS-14), wellbeing (WEMWBS), gender (female vs. male only, due to small *n* of non-binary/other genders), and recency and age of diagnosis. We analysed self-esteem and wellbeing as two separate outcome variables: although there is shared variance between these variables, these concepts can be viewed as distinct, with discriminant validity between the two (Robins et al., 2001; Lyubomirsky et al., 2005), and we aimed to understand how identity contributed to each one uniquely. We thus controlled for wellbeing/self-esteem in the regression analysis predicting the other to ensure that variance explained by these inter-related concepts was taken into account in the model. In step 2, we entered the personal autistic identity variables (the two QDIO subscales: autism pride and exclusion/dissatisfaction) as predictors.

Similarly, we carried out a second hierarchical regression with wellbeing (WEMWBS) as the dependent variable, with the same variables as above controlled for in step 1 (replacing wellbeing with self-esteem), and personal autistic identity (QDIO subscales) in step 2. Together, these analyses aimed to show how personal identity contributed to wellbeing/self-esteem, over and above gender, diagnostic timing, autistic characteristics, and shared variance between wellbeing and self-esteem. All assumptions were met for the regressions, including homoscedasticity and multi-collinearity.

#### Qualitative Analysis

We used content analysis to interpret qualitative responses (Hsieh and Shannon, 2005). One author (KC) condensed responses into meaning units. Codes that were related to each other through content or context were grouped into categories, which were discussed and agreed with a second independent reviewer (EC; agreement 88%), with any disagreements discussed before coding was finalised. Responses could be coded into multiple categories.

In qualitative research, it is essential to acknowledge and discuss the positionality of the researcher (Braun and Clarke, 2019). One of the researchers is an autistic person who received her diagnosis in early adulthood, a process she found immensely challenging at the time, in part due to a lack of post-diagnostic resources. Her subsequent experiences within training for Clinical Psychology have included involvement in service development. These factors led to her interest in designing and conducting this research. High level of agreement with a second reviewer demonstrates validity in the identified categories.

## Results

### Descriptive Statistics

Table 2 shows the means and SDs for the quantitative variables included in the study, and Table 3 includes correlations between the variables.

**Table 2:** Means and SDs for autistic characteristics, self-esteem, wellbeing, and personal identity subscales.

Variable	Mean (SD)	Range
Autistic characteristics	35.47 (4.67)	23–42
Self-esteem	24.97 (4.31)	13–32
Wellbeing	39.54 (6.75)	20–59
Personal identity		
Autistic pride	13.62 (2.34)	9–20
Exclusion/Dissatisfaction	10.87 (2.70)	4–16

**Table 3:** Pearson’s correlations between the variables included in the study.

	Gender	Age of diagnosis	Recency of diagnosis	Autistic characteristics	Self-esteem	Well-being	Autistic Pride
Age of diagnosis	−0.123						
Recency of diagnosis	0.599^***^	−0.399^***^					
Autistic characteristics	−0.045	0.004	0.005				
Self-esteem	0.285^***^	−0.184^*^	0.410^***^	−0.055			
Wellbeing	0.251^**^	−0.195^*^	0.331^***^	−0.083	0.864^***^		
Autistic pride	0.102	−0.036	0.131^*^	0.002	0.592^***^	0.525^***^	
Exclusion/Dissatisfaction	−0.108	0.263^***^	−0.401^***^	0.049	−0.778^***^	−0.738^***^	−0.466^***^

*Gender coded as 0 = female, 1 = male (inclusion of other genders in statistical analyses precluded by small sample size)>*.

*** *p* < 0.001;

** *p* < 0.01;

* *p* < 0.05 (2-tailed).

### Age and Recency of Diagnosis

**Figure 1:**
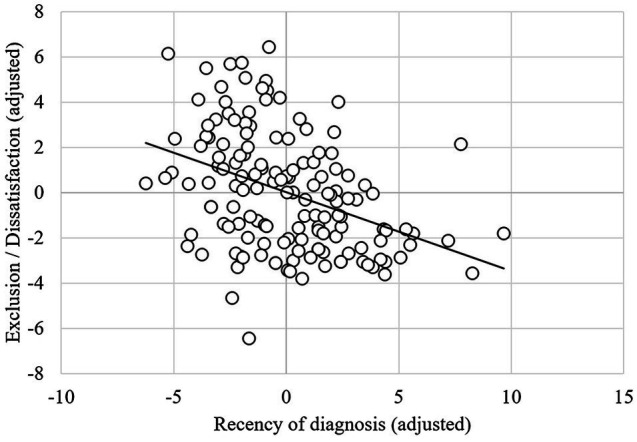
Partial regression plot showing the relationship between recency of diagnosis and exclusion/dissatisfaction (controlling for other variables in the model).

Results from the two multiple regressions, with the personal autistic identity subscales as outcomes and age at and recency of diagnosis as predictors (controlling for gender), are summarised in Table 4. With autism pride as the outcome, the model explained 2.2% of the variance and was not significant [*F*(3,143) = 1.07, *p* = 0.36], with no significant predictors. With exclusion/dissatisfaction as the outcome, the model explained 21.5% of the variance and was significant [*F*(3,143) = 12.07, *p* < 0.001]. Here, as number of years since diagnosis increased, exclusion/dissatisfaction decreased (Figure 1).

**Table 4:** Regression results for the two multiple regressions with each of the Questionnaire on Disability Identity and Opportunity (QDIO) subscales as outcomes variables, and age, recency of diagnosis, and gender as the predictors.

	B	B CI	SE B	*β*	*p*	*f* ^2^
Outcome: Autism pride						
Recency	0.084	[−0.047–0.21]	0.066	0.14	0.21	0.011
Age of diagnosis	0.004	[−0.034–0.041]	0.019	0.017	0.85	0.0003
Gender	0.113	[−1.08–1.30]	0.60	0.019	0.85	0.0002
Outcome: Exclusion/Dissatisfaction					
Recency	−0.35	[−0.49–0.21]	0.069	−0.51	<0.001	0.16
Age of diagnosis	0.024	[−0.015–0.063]	0.020	0.10	0.22	0.008
Gender	1.39	[0.15–2.64]	0.63	0.21	0.028	0.028

B, unstandardised beta coefficient; B CI, confidence intervals at 95% lower and upper bounds; SE B, standard error; β, standardised beta coefficient; *f*^2^, effect size (0.02 considered small effect, 0.15 medium, 0.35 large).

### Self-Esteem

**Figure 2:**
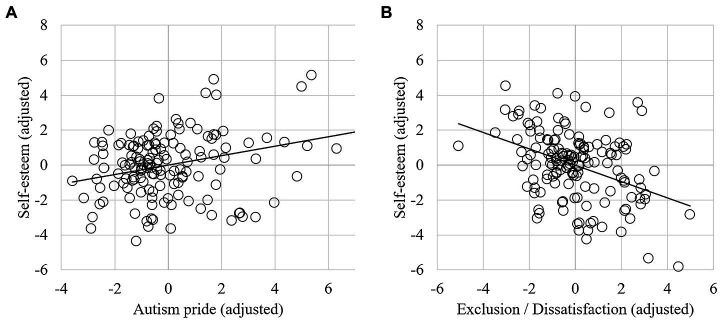
Partial regression plots showing the relationship between self-esteem and **(A)** “autism pride” and **(B)** “exclusion/dissatisfaction” (controlling for other variables in the model).

The first step, including wellbeing, autistic characteristics, gender, and recency and age of diagnosis explained 77.1% of the variance in self-esteem (Table 5), and the model was significant [*F*(5, 141) = 95.21, *p* < 0.001]. Adding the QDIO subscales into step 2 explained an additional 5.7% of the variance in self-esteem, which was a significant increase [*F*(2, 139) = 22.84, *p* < 0.001]. The final model was significant [*F*(7,139) = 95.61, *p* < 0.001] and explained 82.8% of the variance in self-esteem. Specifically, lower exclusion/dissatisfaction significantly predicted higher self-esteem, and greater autism pride predicted higher self-esteem (Figure 2). Wellbeing was also a significant predictor in the final model, such that higher wellbeing predicted higher self-esteem. No other variables were significant.

**Table 5:** Hierarchical regression results with self-esteem as the outcome variable.

Predictor	B	B CI	SE B	β	*p*	*f* ^2^
Step One						
Wellbeing	0.53	[0.47–0.58]	0.028	0.83	<0.001	1.34
Autistic characteristics	−0.014	[−0.089–0.061]	0.038	−0.015	0.72	0.0002
Gender	−0.03	[−1.11–1.05]	0.55	−0.003	0.96	0.000004
Recency of diagnosis	0.16	[0.034–0.28]	0.062	0.14	0.013	0.011
Age of diagnosis	0.014	[−0.019–0.048]	0.017	0.037	0.40	0.001
Step Two						
Wellbeing	0.35	[0.27–0.42]	0.036	0.54	<0.001	0.13
Autistic characteristics	−0.016	[−0.082–0.050]	0.033	−0.017	0.63	0.00002
Gender	0.77	[0.23–1.76]	0.502	0.071	0.13	0.002
Recency of diagnosis	0.065	[−0.048–0.18]	0.057	0.059	0.26	0.001
Age of diagnosis	0.015	[−0.014–0.045]	0.015	0.040	0.30	0.001
Autism pride	0.27	[0.12–0.43]	0.079	0.15	<0.001	0.015
Exclusion/Dissatisfaction	−0.47	[−0.65–−0.29]	0.092	-0.29	<0.001	0.033

### Wellbeing

**Figure 3:**
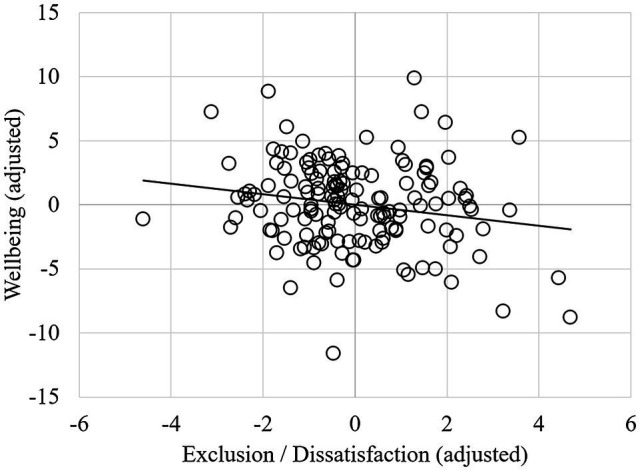
Partial regression plot showing the relationship between wellbeing and exclusion/dissatisfaction (controlling for other variables in the model).

The first step, including self-esteem, gender, autistic characteristics, and recency and age of diagnosis, explained 75.9% of the variance in wellbeing scores (Table 6), and the model was significant [*F*(5, 141) = 89.01, *p* < 0.001]. Adding the QDIO subscales as predictors explained an additional 0.9% of the variance in wellbeing, which was not a significant increase [*F*(2, 139) = 2.84, *p* = 0.062]. The final model significantly explained 76.9% of the variance in wellbeing scores [*F*(7,139) = 66.05, *p* < 0.001], with greater exclusion/dissatisfaction predicting lower wellbeing at a suggestively significant threshold (Figure 3). As before, higher self-esteem also predicted higher wellbeing, and no other variables were significant in the final model.

**Table 6:** Hierarchical regression results, with wellbeing as the outcome variable.

Predictor	B	B CI	SE B	β	*p*	*f* ^2^
Step One						
Self-esteem	1.36	[1.22–1.50]	0.072	0.87	<0.001	1.52
Autistic characteristics	−0.024	[−0.15–0.097]	0.061	−0.016	0.70	0.0003
Gender	0.028	[−1.47–2.02]	0.88	0.016	0.76	0.0002
Recency of diagnosis	−0.058	[−0.26–0.14]	0.10	−0.034	0.57	0.0006
Age of diagnosis	−0.034	[−0.088–0.020]	0.027	−0.056	0.21	0.003
Step Two						
Self-esteem	1.14	[0.90–1.38]	0.12	0.73	<0.001	0.18
Autistic characteristics	−0.026	[−0.15–0.093]	0.060	−0.018	0.67	0.0003
Gender	0.95	[−0.86–2.76]	0.92	0.056	0.30	0.002
Recency of diagnosis	−0.114	[−0.32–0.093]	0.10	−0.066	0.28	0.002
Age of diagnosis	−0.027	[−0.081–0.027]	0.027	−0.045	0.32	0.002
Autism Pride	0.068	[−0.23–0.36]	0.15	0.023	0.65	0.0004
Exclusion/Dissatisfaction	−0.41	[−0.77–−0.059]	0.18	−0.17	0.023	0.009

### Qualitative Analyses

Fifty-four participants (35.7% of the sample) responded to the optional qualitative question, describing how receiving an autism diagnosis impacted on their thoughts and feelings about themselves. Table 7 illustrates the identified categories and sub-categories, number of coded responses for each category, and example quotes.

**Table 7:** Categories and sub-categories identified in relation to the qualitative question about receiving an autism diagnosis.

Categories	N	Example quotes
Adjustment process		
Making sense of it all over time	56	*“[The diagnosis] led me to re-evaluate my life;” “It changed everything. Things finally made sense.”*
Emotional reaction to diagnosis	39	*“I felt relieved;” “I was upset, confused and angry.”*
Permanence of difficulties	12	*“Sometimes now I feel a bit sad about my autism because I know I am always going to find things difficult;” “Confirmed I was different and would never be normal.”*
Self-exploration		
Knowing and understanding who I am	77	*“[It’s] good to know why I felt different;” “I think probably the diagnosis has helped me more than not as I know myself better.”*
Being myself	10	*“I give myself space to be me;” “[I’m] letting myself be the real me.”*
Feeling the same about myself	7	*“[The] diagnosis only confirmed what I felt about myself;” “I’m still not a very confident person really.”*
Learning and support needs		
I’ve learned a lot	24	*“It took time, but I read around a lot, learned online…”; “Mostly I’ve learned from other people online about how to help myself.”*
More support needed post-diagnosis	13	*“It was frustrating that there was nobody to help me through;” “They did not really tell me very much about my diagnosis, so for years I thought that being autistic meant having meltdowns and being crap at human interaction and that was pretty much it.”*
Late identification	10	*“Why had not it [autism] been picked up sooner?;” “It was a double edged sword though as it also caused anger since no one has noticed for so long.”*
Autism in women	6	*“People do not understand autism in girls;” “A friend mentioned something about women with autism, which prompted me to do loads of research and reading. I was amazed!”*
Responses from others		
Lack of understanding/acceptance from others	30	*“People said my diagnosis is fake;” “People do not understand autism.”*
Issues with disclosure	6	*“I’m still reluctant to tell people I’m autistic;” “2020s still not safe to be openly autistic as a professional person.”*
Positive reactions	6	*“I felt seen, heard and understood;” “It helped my family understand too.”*
Autism as positive difference		
Autistic pride and appreciation	18	*“[I’m] learning to be proud of my autism;” “Autism is an attribute.”*
Thinking differently/being different	13	*“This is how my brain works;” “I’ve fully embraced my neurology.”*
Self-advocacy	6	*“[I’m] more confident asking for accommodations;” “By being able to advocate for my needs, discrimination that happened in the past happens less after the initial denial of services.”*
Challenges of the diagnosis		
Struggling to come to terms and find my place	29	*“I was very self-conscious and lost confidence;” “Sometimes it feels lonely. I do not always feel like I have much in common with other autistic people, so I’m not always sure where I fit in or belong”*
Autism as a negative	8	*“It felt very negative at first, like something was broken that could not be fixed;” “I also had quite negative views about autism and felt like people were insulting me when they said that I was very likely to be autistic”*
*I now feel part of something with others like me*	*15*	*“Other people go through the same;” “[I’m] less alone.”*

The most reported category focused on an “adjustment process,” with responses generally reflecting changes experienced by the respondents following diagnosis. Within this category, we identified three sub-categories. First, participants discussed “Making sense of it all over time” whereby they referred to a process of re-evaluating their lives following their diagnosis. Further, participants talked about an “Emotional reaction to diagnosis,” describing various reactive emotional responses including both positive (such as relief and happiness) and negative feelings (such as confusion and being overwhelmed). Additionally, some participants discussed “Permanence of difficulties,” conveying a sense of grief or frustration over having to accept that their difficulties would not go away.

The next most reported category concerned “Self-exploration,” referring to the experience or process of self-discovery following diagnosis. There were three sub-categories: most often, participants discussed “Knowing and understanding who I am,” highlighting how they had an increased understanding of themselves after diagnosis, in addition to allowing themselves more self-compassion. Some participants discussed “Being myself,” describing feeling increasingly able to be more authentic in themselves. However, some participants discussed “Feeling the same about myself,” reflecting the sense that the diagnosis had not led to any change in their self-perceptions.

Within the category of “Learning and support needs,” participants reflected on a lack of information or knowledge about autism following diagnosis: Within the sub-category “I’ve learned a lot,” participants mentioned their own preconceptions about autism before and after receiving the diagnosis and discussed finding out more about autism by doing research following diagnosis. The sub-category “More support needed post-diagnosis” referred to a perceived lack of post-diagnostic support and services available. A few participants mentioned “Late identification,” questioning how it had taken until adulthood for others to recognise them as autistic. Relatedly, some participants specifically mentioned “Autism in women,” describing a specific lack of information available about autistic females.

The next category focused on “Responses from others,” referring to the reactions (both positive and negative) from other people to the respondent’s diagnosis. Here, within the sub-category “Lack of understanding and acceptance from others,” participants discussed difficult interactions they had with others on receiving their diagnosis, particularly in managing others’ poor autism knowledge and being disbelieved or not accepted by others. Some discussed the sub-category of “Issues with disclosure,” reporting concerns about disclosing their diagnosis to others. A few participants described “Positive reactions,” giving examples of others responding positively to the diagnosis, often in terms of increased understanding.

In the category “Autism as a positive difference,” responses included descriptions of positive aspects of receiving a diagnosis. Here, within the sub-category “Autistic pride and appreciation,” participants discussed the strengths associated with being autistic, in addition to experiencing a positive autistic identity. Some participants mentioned “Thinking differently/being different” where they discussed positive aspects of difference and described the diagnosis as replacing a previous sense of something being “wrong” with themselves. A few participants discussed the sub-category of “Self-advocacy,” reflecting on an increased ability to advocate for themselves and their needs following diagnosis.

Less often, we coded responses into the category “Challenges of the diagnosis,” where participants reflected on negative aspects of receiving a diagnosis. Within the sub-category “Struggling to come to terms and find my place,” participants reflected on feelings of low self-confidence and not relating to their pre-existing perceptions of autism. We coded a few responses as “Autism as a negative,” where participants described a sense of negative difference or focused on difficulties associated with being autistic.

The final category, “I now feel part of something with others like me,” reflected a sense of connection with the autistic community, how receiving an autism diagnosis helped participants to feel less lonely, and provided a sense of hope that there were other people who have similar experiences.

## Discussion

The current study sought to understand aspects of identity, self-esteem, and wellbeing in relation to an autism diagnosis in adulthood. We found that with greater number of years since diagnosis (but not age of diagnosis), participants reported less exclusion/dissatisfaction with being autistic. Our results also indicated that greater dissatisfaction with being autistic related to lower self-esteem, and higher pride in being autistic related to greater self-esteem. Greater dissatisfaction also related to poorer wellbeing, although this finding is treated with caution. Qualitative responses reflected a process of cognitive and emotional reaction to receiving a diagnosis and gave a sense of learning and change over time, supporting our quantitative findings. The qualitative data also elucidated other psychological processes following diagnosis in terms of self-exploration, social connection, and support-seeking.

Contrary to our hypothesis, only recency of diagnosis predicted exclusion/dissatisfaction, with participants feeling more satisfied with being autistic as years passed following diagnosis. These findings could indicate that receiving a diagnosis offers a growing awareness of “being autistic,” and as such, a sense of exoneration in explaining the underlying basis of a person’s strengths and difficulties over time (Punshon et al., 2009). Indeed, qualitative work with autistic people suggests that the navigation of stigma, stereotypes, and discrimination can be exceptionally challenging for autistic people when they conceptualise their identity (Botha et al., 2020). Our findings also support qualitative research with older late-diagnosed autistic adults, some of whom appeared to externalise and reject autism as part of their identity (Hickey et al., 2018). Over time, increasing self-identification as autistic following diagnosis may encourage a view of autism as a positive difference instead of a deficit (Kapp et al., 2013). Our findings fit with other surveys where autistic adults reported negative emotions after having their diagnosis confirmed (Jones et al., 2014), and qualitative studies highlighting “painful” adjustments following diagnosis that eventually leads to greater self-compassion (Leedham et al., 2020). As time passes, autistic people may also feel better equipped to self-advocate and challenge pre-existing stereotypes (Botha et al., 2020). Our qualitative data included references to a learning process following diagnosis, whereby previously held stereotypes or misconceptions about autism were challenged in favour of more positive views. This finding would fit with studies that have shown how learning about autism and neurodiversity helps with the development of a more holistic conception of autism (King et al., 2003; Griffin and Pollak, 2009).

Due to the cross-sectional nature of the present study, it is only possible to theorise about identity mechanisms and other factors. A longitudinal design would be necessary to determine how an autistic personal identity develops over time, as well as identifying potential individual differences in trajectories. Our findings suggest that autistic personal identity varies over time following diagnosis, with more negative elements initially endorsed, and this dissatisfaction may decrease over time. The initial agreement with negative elements post-diagnosis is supported by theories of identity development which account for an initial disruptive impact of “acquiring” a condition or disability (Frank, 1993). Within this theory, adapting to a diagnosis leads to a period of critical reflection (Charmaz, 1994, 1995), with self-change necessary to accommodate disability into one’s identity.

Becoming more satisfied with autistic identity could also be understood in terms of undergoing a period of adjustment. This finding would fit with the concept of “identity distress,” which relates to the existential anxiety and maladjustment associated with amalgamating a coherent set of beliefs about one’s identity (Berman et al., 2004). Further, an analysis of wellbeing in autistic adults identified narratives describing a personal journey from hating “their autism” to seeing themselves as a “person with autism,” to an “autistic person” (Milton and Sims, 2016). Indeed, participants who responded qualitatively about their experiences in the present study also described an adjustment process including changes in cognitive and emotional responses, alongside increased knowledge and understanding of autism overall, and in relation to themselves.

Regarding our second hypothesis, we found that greater exclusion/dissatisfaction with being autistic predicted both lower self-esteem and poorer wellbeing, controlling for other variables, such as demographics, diagnostic timing, and autistic characteristics. This finding suggests that irrespective of exactly when someone is diagnosed, identity is an important variable in self-esteem and wellbeing. The exclusion/dissatisfaction subscale represented negative beliefs about autism being part of one’s personal identity, relating to perceived limitations on social life, work, and quality of life (Darling and Heckert, 2010). In this way, autism pride and exclusion/dissatisfaction act as opposing sides of the same construct within autistic personal identity. Of interest, however, is that exclusion/dissatisfaction only suggestively predicted wellbeing (with a very small effect size), and autism pride did not predict wellbeing at all. Botha and Frost (2020) outline how autistic individuals are a minority group, subject to stigma and disadvantage. Their study found that minority stressors, such as discrimination, internalised stigma, and concealment predicted poorer mental health and wellbeing. Therefore, aspects of exclusion/dissatisfaction could bear more similarity to internalised stigma, which could thus have a more negative relationship with self-esteem. Additionally, there may have been weaker relationships with wellbeing as the measure may not have captured autistic wellbeing accurately (Lam et al., 2021). Wellbeing may also be more subjective and multi-dimensional than self-esteem (Rosenberg et al., 1995).

Nonetheless, higher feelings of pride in personal autistic identity predicted higher levels of self-esteem. This relationship may be explained when considering how pride involves self-acceptance and self-compassion, which are similar to constructs underlying self-esteem, such as optimism and self-satisfaction (Luhtanen and Crocker, 1992). The finding could also reflect connections between personal and social identities, which likely influence one another. Cooper et al. (2017) suggested that autistic social identity (i.e., identifying with other autistic people as a group) involves not only a connection to the autistic community, but also internalisation of this social identity within one’s self-concept. They found that an autistic social identity related to greater self-esteem, and our findings develop this finding by showing that one’s personal sense of autistic pride (i.e., “being autistic is an important part of who I am”) also relates to higher self-esteem. Additionally, qualitative responses indicated a desire for information and connectedness, which may link to engagement with the autistic community. Indeed, a study of diagnosis disclosure in autistic adolescents found that youth who sought information and support from other autistic people reported better outcomes in self-esteem and diagnosis acceptance than those who learned about autism from other sources (Kiely et al., 2020). Since age and recency of diagnosis were controlled for in all our analyses, and did not significantly contribute to self-esteem or wellbeing, this suggests that autistic identification, rather than diagnosis/recognition itself, might be especially important when it comes to psychological health (although recognition is a step to identification).

### Implications

This study particularly highlights the experiences of autistic people who were diagnosed late, and demonstrates relationships between autistic personal identity, self-esteem, wellbeing, and diagnostic timing. Self-esteem and wellbeing can both be understood as closely related to broader psychological health and functioning (Lyubomirsky et al., 2005). Although the correlational nature of the current study design inhibits the ability to determine causation, our results suggest that exploring potential identity-based support for psychological health should be a priority for future research. In the disability literature, it has been recommended that disabled people should be encouraged to engage with their personal disability narrative to aid the development of coping strategies and positive identity development (Dunn and Burcaw, 2013). For autistic people, this could involve a programme supporting newly diagnosed autistic people to think about strengths, challenges, and fostering connections with the wider autistic community. A recent study has highlighted the specific benefits of such group programmes being autistic-led in terms of developing a positive outlook on being autistic (Crane et al., 2020), and research highlights the benefits of autistic-autistic peer communication (Crompton et al., 2020a,b).

Further, Forber-Pratt et al. (2019) suggest that professionals are often the most significant or accessible resource for many following diagnosis, and therefore have a role to play in influencing people’s relationship with their autistic identity. However, some participants in the present study noted that lack of post-diagnostic professional support was an issue, which has been reported previously – with 42% of respondents in one survey not offered any type of post-diagnostic support (Jones et al., 2014). Although it is reassuring that many of the participants in this study connected with other autistic people online for information and support, the current findings suggest there is a need for services and clinicians to provide higher quality post-diagnostic support.

### Limitations

There were several limitations with our sample: only four participants identified as either non-binary or transgender, which was too few to include in statistical analyses. This limitation restricted the quantitative analyses to cisgender participants and is particularly relevant given increased gender identity variance in autistic individuals (De Vries et al., 2010; Pasterski et al., 2014). Further, participants were predominantly female (77.6%), suggesting a potential issue in terms of overall representativeness of the female experience. Since there has been a systematic under-identification of autism in females, with females at higher risk of being misdiagnosed or diagnosed late (Mandy et al., 2012; Kreiser and White, 2014; Trubanova et al., 2014), partly due to the male-biased development of assessment measures and diagnostic criteria (Kreiser and White, 2014; Loomes et al., 2017), the study may have been of particular interest to females. Given the challenges faced by autistic females in terms of unmet support needs, social exclusion, and isolation (Baldwin and Costley, 2016), our study indicates that understanding and supporting autistic females to explore their autistic identity may be beneficial. Nonetheless, future research should aim to establish whether the current findings are replicable in a larger sample of males.

The sample was also predominantly white, and university-educated. However, the demographic figures reported in the present study are broadly similar to comparable survey research (e.g., Cooper et al., 2017; Cage and Troxell-Whitman, 2019). Lack of diversity is a frequent criticism of autism literature (Pellicano et al., 2014). However, the sampling of autistic adults and particularly females builds upon a previous lack of representation in autism research (Kirkovski et al., 2013; Pellicano et al., 2014). Further, this study utilised convenience sampling through social media and groups, which may have led to biases in the sample. For example, in a study of autistic adults’ participation in research, factors such as altruism, a sense of community, and a keenness to be listened to and understood were found to be particularly motivating (Haas et al., 2016). Additionally, our sample is biased in that it consisted of mostly well-educated individuals recruited *via* the internet, and this sample will not be representative. Our findings represent only a subset of autistic people, and although there was variation in autistic identification, autistic pride was generally high. Future research should attempt to recruit participants from a wider variety of online and offline sources and find ways to capture the views of the autistic community more broadly. Finally, the measure of personal autistic identity was adapted from the disability literature (Darling and Heckert, 2010), and may not have fully captured a personal autistic identity. The development of measures specifically to capture the incorporation of “autism” into personal identity would thus be beneficial. The constructs of wellbeing and self-esteem were also based on measures that have been created by and validated with non-autistic people.

### Conclusion

The present study explored the relationships between autistic personal identity, diagnostic timing, and psychological health, with a focus on late-diagnosed autistic people. With more time, since diagnosis there was less dissatisfaction with being autistic and autism pride and exclusion/dissatisfaction significantly predicted self-esteem, and exclusion/dissatisfaction suggestively predicted wellbeing. Qualitative descriptions of diagnosis experiences described a post-diagnostic process that included emotional reactions and self-exploration, which developed into self-acceptance and belonging. Our results add to the literature concerning the experiences of late-diagnosed autistic adults, with implications regarding the need for more frequent and comprehensive provision of information and post-diagnostic support and finding ways to enable all autistic people to explore their autistic personal identity.

## Data Availability Statement

The raw data supporting the conclusions of this article will be made available following reasonable request to the corresponding author.

## Ethics Statement

The studies involving human participants were reviewed and approved by Research Ethics Committee, Royal Holloway, University of London. The participants provided their informed consent to participate in this study.

## Author Contributions

KC conceived the research and managed participant recruitment with the support of EC and RB. All authors contributed to study design. KC and EC analysed the quantitative and qualitative data. KC wrote the first draft of the manuscript as a chapter for her thesis, and EC was a major contributor to subsequent drafts. RB further contributed to manuscript drafts. All authors contributed to the article and approved the submitted version.

## Conflict of Interest

The authors declare that the research was conducted in the absence of any commercial or financial relationships that could be construed as a potential conflict of interest.

## Publisher’s Note

All claims expressed in this article are solely those of the authors and do not necessarily represent those of their affiliated organizations, or those of the publisher, the editors and the reviewers. Any product that may be evaluated in this article, or claim that may be made by its manufacturer, is not guaranteed or endorsed by the publisher.
